# Species-Specific Activation of TLR4 by Hypoacylated Endotoxins Governed by Residues 82 and 122 of MD-2

**DOI:** 10.1371/journal.pone.0107520

**Published:** 2014-09-09

**Authors:** Alja Oblak, Roman Jerala

**Affiliations:** 1 Department of Biotechnology, National Institute of Chemistry, Ljubljana, Slovenia; 2 Centre of Excellence EN-FIST, Ljubljana, Slovenia; McGill University, Canada

## Abstract

The Toll-like receptor 4/MD-2 receptor complex recognizes endotoxin, a Gram-negative bacterial cell envelope component. Recognition of the most potent hexaacylated form of endotoxin is mediated by the sixth acyl chain that protrudes from the MD-2 hydrophobic pocket and bridges TLR4/MD-2 to the neighboring TLR4 ectodomain, driving receptor dimerization via hydrophobic interactions. In hypoacylated endotoxins all acyl chains could be accommodated within the binding pocket of the human hMD-2. Nevertheless, tetra- and pentaacylated endotoxins activate the TLR4/MD-2 receptor of several species. We observed that amino acid residues 82 and 122, located at the entrance to the endotoxin binding site of MD-2, have major influence on the species-specific endotoxin recognition. We show that substitution of hMD-2 residue V82 with an amino acid residue with a bulkier hydrophobic side chain enables activation of TLR4/MD-2 by pentaacylated and tetraacylated endotoxins. Interaction of the lipid A phosphate group with the amino acid residue 122 of MD-2 facilitates the appropriate positioning of the hypoacylated endotoxin. Moreover, mouse TLR4 contributes to the agonistic effect of pentaacylated msbB endotoxin. We propose a molecular model that explains how the molecular differences between the murine or equine MD-2, which both have sufficiently large hydrophobic pockets to accommodate all five or four acyl chains, influence the positioning of endotoxin so that one of the acyl chains remains outside the pocket and enables hydrophobic interactions with TLR4, leading to receptor activation.

## Introduction

Immediate and effective innate immune response is crucial for the prevention of microbial colonization or dissemination in mammalian host. The defensive actions are enabled by innate immune receptors that sense conserved microbial structures and trigger immediate response by the cells of the immune system. Arguably the most important and so far the most studied group of innate immune receptors are the Toll-like receptors (TLRs). They specifically recognize distinctive microbial molecules, such as double stranded RNA, flagellin or endotoxins [1].

Endotoxins (*i.e.* lipopolysaccharides, LPS) are the main constituents of the cell envelope of most Gram-negative bacteria (with few exceptions [2]–[4]). They are composed of a highly variable polysaccharide and a lipid A moiety, which is the active principle of endotoxin that is recognized by the TLR4/MD-2 receptor complex. Lipid A is typically composed of a glucosamine disaccharide modified with two phosphates and comprising four primary glucosamine-linked hydroxyacyl chains and two secondary acyl chains [5]. This type of hexaacylated endotoxin can be found in the majority of Gram-negative bacteria, ranging from soil and plant inhabitants to mammalian mucosal pathogens. The hexaacylated endotoxin activates the human TLR4/MD-2 very efficiently, so it is not surprising that some bacteria modify their lipid A structure in order to evade immune recognition [6]. *Pseudomonas aeruginosa* [7] can produce weakly activating pentaacylated endotoxin and *Yersinia pestis* and *Helicobacter pylori* can produce a tetraacylated form of lipid A [8], [9], which cannot activate the human TLR4/MD-2, and can thus avoid activation of the immune response.

Hypoacylated forms of endotoxin are also precursors in the bacterial synthesis of lipid A (*i.e.* the tetraacylated lipid IVa that has only the primary hydroxyacyl chains) or can be the result of mutations in genes such as *msbB*, which is a gene in the *E. coli* endotoxin synthesis pathway that encodes an acyltransferase. Mutation in *msbB* results in production of a pentaacylated endotoxin that lacks a secondary myristoyl fatty acid [10], [11]. *MsbB* homologues have been characterized in a variety of pathogenic bacteria (*e.g., Salmonella* spp., *Neisseria* spp., *Yersinia pestis*, *Klebsiella pneumoniae*) [12]–[15]. Moreover, a second functional paralog of the *msbB* gene was identified in *Shigella* and enterohemorrhagic *E. coli* [16]–[19].

While hypoacylated endotoxins antagonize the activation of human TLR4/MD-2 complex by hexaacylated lipid A, they potently activate the TLR4/MD-2 receptor of several other species (*e.g.,* mouse, rat, horse) [20]–[22]. The molecular difference in TLR4/MD-2 responsible for this discriminating endotoxin recognition is still not clear. Several studies have analyzed the molecular differences in the structure of MD-2 and TLR4 between species and identified regions in the endotoxin receptor complex that are involved in this discrimination [21], [23]–[25].

In the present study we further unravel the structural differences between human, mouse and equine MD-2 that are important for species-specific recognition of endotoxin varieties. We provide evidence that amino acid residues 82 and 122 crucially influence the positioning of endotoxin into MD-2′s hydrophobic pocket and therefore govern the agonistic/antagonistic properties of endotoxin varieties in different species. We were able to confer human MD-2 responsiveness to tetra- and pentaacylated LPS through increasing the hydrophobicity at the edge of the MD-2 binding pocket.

## Materials and Methods

### Reagents

Expression plasmids containing sequences of human TLR4 and MD-2 as well as the pELAM-1 firefly luciferase plasmid were a gift from Dr. C. Kirschning (Technical University of Munich, Germany). Expression plasmid containing the sequence of mouse TLR4 was purchased from InvivoGen (CA, USA). Expression plasmid for mouse MD-2 was a gift from Dr. Y. Nagai (University of Tokyo, Japan). Expression plasmid for horse (equine) MD-2 and TLR4 were a gift from Dr. M. Vandenplas (University of Georgia, USA). The Renilla luciferase phRL-TK plasmid was purchased from Promega (WI, USA). Synthetic hexaacylated lipid A (compound 506) and tetraacylated lipid IVa (compound 406) were purchased from Peptide Institute (Japan). The pentaacylated msbB endotoxin was purchased from InvivoGen (CA, USA). All nucleotide sequences encoding MD-2 were cloned into pEF-BOS vector with Flag and His tags on the C-terminal. All nucleotide sequences encoding TLR4 were cloned into pUNO vector with C-terminal HA tag. Transfection reagent JetPEI was purchased from Polyplus-Transfection (France) and was used according to the manufacturer's instructions.

### Cell cultures

The human embryonic kidney (HEK) 293 cells were kindly provided by Dr. J. Chow (Eisai Research Institute, Andover, USA). Flp-In T-REx cells and the Flp-In system were purchased from Invitrogen (CA, USA). T-REx cell lines stably expressing human or mouse TLR4 were made using the Flp-In system according to the manufacturer's instructions. Briefly, human and mouse TLR4 nucleotide sequences were cloned from the pUNO-HA vector into the pcDNA5/FRT expression vector, which was then transfected into Flp-In T-REx cells along with pOG44 expression plasmid for expression of the Flp recombinase. After homologous recombination between the FRT recombination sites in the T-REx cell genome and the pcDNA5/FRT vector, stable cells were selected for hygromycin resistance. All cells were grown in DMEM supplemented with 10% FBS.

### Site-directed mutagenesis

All MD-2 mutants were made using QuikChange site-directed mutagenesis kit (Stratagene, USA) according to the manufacturer's instructions. All plasmids were sequenced to confirm the mutation.

### Cell activation assay – NF-κB-luciferase reporter assay

HEK293 or HEK293/TLR4 (T-REx) cells were seeded in 96-well Costar plates (Corning, NY, USA) at 3,5·10^4^ cells/well and incubated overnight in a humidified atmosphere (5% CO_2_) at 37°C. The next day, when cells were 60–80% confluent, they were co-transfected with pEF-BOS-MD-2 (10 ng), NF-κB-dependent luciferase (50 ng) and constitutive Renilla (10 ng) reporter plasmids and (in the case of HEK293 cells) pUNO-TLR4 plasmid (1 ng) using JetPEI transfection reagent. Cells were stimulated 6 hours after transfection with endotoxin preparations that were extensively vortexed immediately prior to stimulation. Cells were lysed after 16 hours of stimulation in 1× reporter assay lysis buffer (Promega, USA) and analyzed for reporter gene activities using a dual-luciferase reporter assay system. Relative luciferase units (RLU) were calculated by normalizing each sample's luciferase activity for constitutive Renilla activity measured within the same sample. When plotting the data the value of the wild type TLR4/MD-2 sample stimulated with lipid A was normalized to 100 and other values were adjusted accordingly. Experiments were independently performed at least three times with similar results, each time in at least three replicates. Figures show results from a representative experiment with standard deviation. The p values were calculated using Student one-tailed t-test.

### ELISA

Human IL-8 concentrations were determined in the supernatants of HEK293 cells. Cells were seeded at 3,5·10^4^ cells/well and incubated overnight. The next day they were co-transfected with plasmids encoding the wt MD-2 or MD-2 mutants (30 ng) and TLR4 (3 ng) using JetPEI transfection reagent and stimulated with the indicated amount of endotoxin. After 16 hours, the supernatants were harvested and the amount of human IL-8 was determined using ReadySetGo ELISA kit (e-Bioscience).

### Molecular structure visualization

Molecular graphics and analyses were performed with the UCSF Chimera package (version 1.6.2). Chimera is developed by the Resource for Biocomputing, Visualization, and Informatics at the University of California, San Francisco [26]. The representation of the MD-2 structures are based on the crystal structures determined by Park et al. [27] (pdb id 3FXI) and Ohto et al. [28] (pdb id 2E56 and 2E59). The model of the MD-2 mutants was prepared with the UCSF Chimera. The calculations of the volumes of the MD-2 binding pockets were performed using CASTp [29].

## Results

### Recognition of hypoacylated endotoxins is species-dependent

While all known species with functional TLR4/MD-2 receptors recognize and are activated by the hexaacylated endotoxin, hypo- or hyperacylated forms of endotoxin activate only cells of certain species. Both MD-2 and TLR4 contribute to this recognition [25], [30]. We systematically compared MD-2 and TLR4 and their combinations from three species, *i.e.* human, mouse and horse, for their ability to recognize and trigger activation by tetraacylated (lipid IVa) and pentaacylated (msbB) endotoxins.

**Figure 1:**
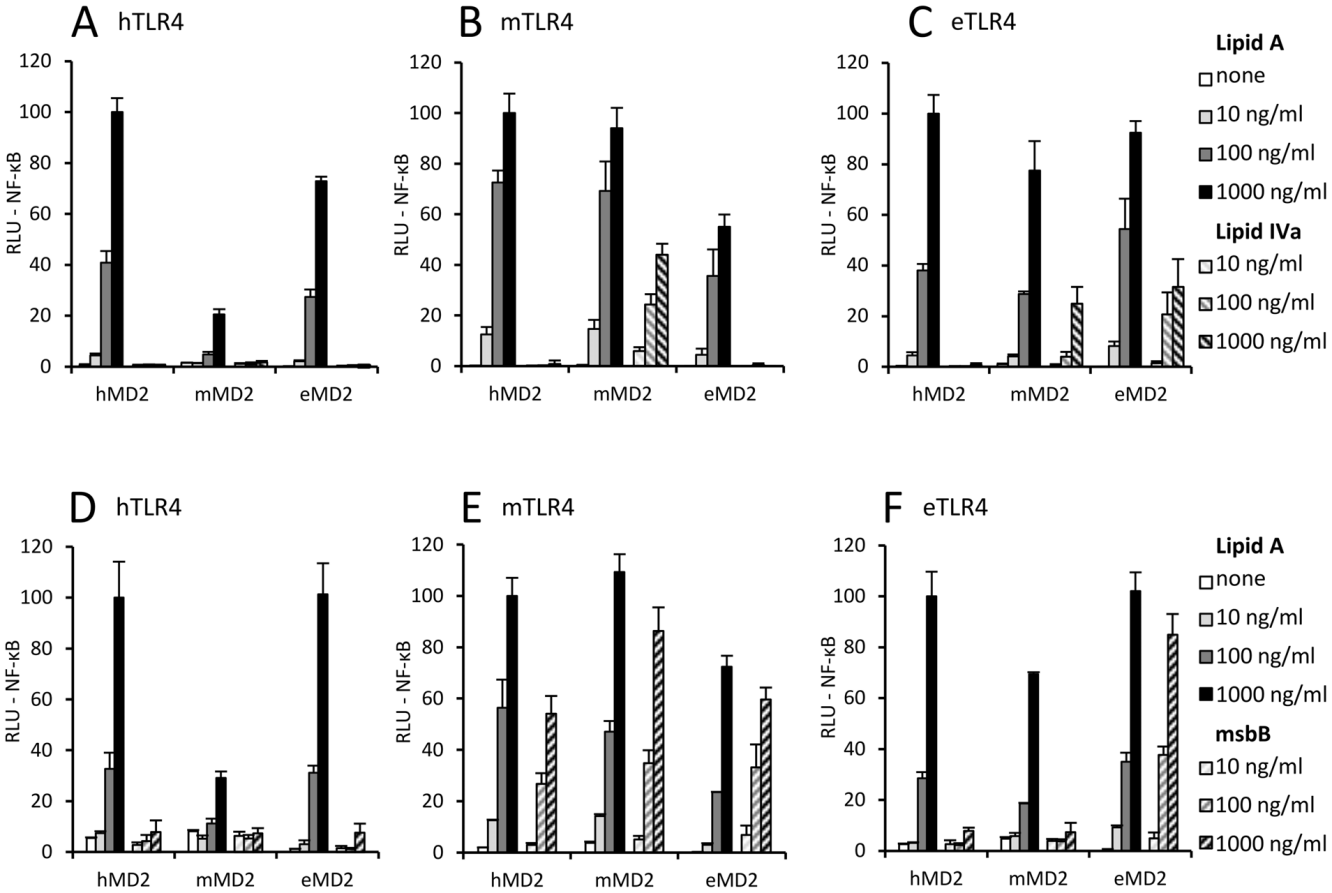
MD-2 supports activation by hypoacylated endotoxins in a species-dependent manner. (A) Activation of human TLR4 by tetraacylated lipid IVa. (B) Murine TLR4 responsiveness to tetraacylated endotoxin. (C) Activation of equine TLR4 by tetraacylated endotoxin. (D–F) Murine TLR4 is best adapted for the responsiveness to pentaacylated msbB endotoxin. (D) Cells expressing human TLR4 do not respond to pentaacylated endotoxin stimulation. (E) Murine TLR4-expressing cells robustly respond to pentaacylated endotoxin regardless of the origin of MD-2. (F) Only equine TLR4/MD-2 complex can support activation by pentaacylated endotoxin.

We show that human TLR4 can be potently activated with hexaacylated lipid A in combination with either human or equine MD-2 (Fig. 1A). None of the MD-2 varieties tested conferred human TLR4 activation by tetraacylated lipid IVa. Mouse TLR4/MD-2 complex is on the other hand potently activated by tetraacylated lipid IVa (Fig. 1B). This activation seems to depend more on mouse MD-2 than on mouse TLR4, since equine TLR4 in combination with mouse MD-2 also supports activation by lipid IVa. Moreover, equine TLR4 appears to be most adept at recognizing lipid IVa, since it can be activated in combination with either equine or mouse MD-2 (Fig. 1C). On the other hand human MD-2 cannot mediate activation by lipid IVa in combination with any TLR4 species. Tetraacylated lipid IVa can therefore activate the mouse TLR4/MD-2 complex as well as the equine TLR4 in combination with either mouse or equine MD-2.

Further on we examined the TLR4/MD-2 receptor activation by the pentaacylated endotoxin msbB. Though lacking only one acyl chain in comparison to the “canonical” hexaacylated endotoxins of enteric bacteria, msbB failed to activate human TLR4 regardless of the MD-2 species used (Fig. 1D–F). TLR4/MD-2 receptor complexes from other species again proved to be less selective, since msbB potently activated both mouse and equine TLR4/MD-2 complex. Contrary to the activation by lipid IVa, where equine TLR4 is better adapted for recognition, mouse TLR4 supported stronger activation by msbB. Mouse TLR4 could mediate msbB-triggered NF-κB activation regardless of the MD-2 species, including human MD-2. On the other hand, equine TLR4 could only be activated with msbB in combination with equine MD-2.

Taken together, the results in figure 1 support the notion that both partners in the TLR4/MD-2 receptor complex contribute to the recognition of hypoacylated endotoxin varieties (Table. 1).

**Table 1:** Responsiveness to different types of endotoxins by combinations of MD-2 and TLR4.

506			
hexaacyl lipid A	hMD-2	mMD-2	eMD-2
hTLR4	+	−	+
mTLR4	+	+	+
eTLR4	+	+	+

406			
tetraacyl lipid A	hMD-2	mMD-2	eMD-2
hTLR4	−	−	−
mTLR4	−	+	−
eTLR4	−	+	+

msbB			
pentaacyl lipid A	hMD-2	mMD-2	eMD-2
hTLR4	−	−	−
mTLR4	+	+	+
eTLR4	−	−	+

### Amino acid residues at positions 82 and 122 of MD-2 are crucial for the species dependent recognition of lipid Iva

**Figure 2:**
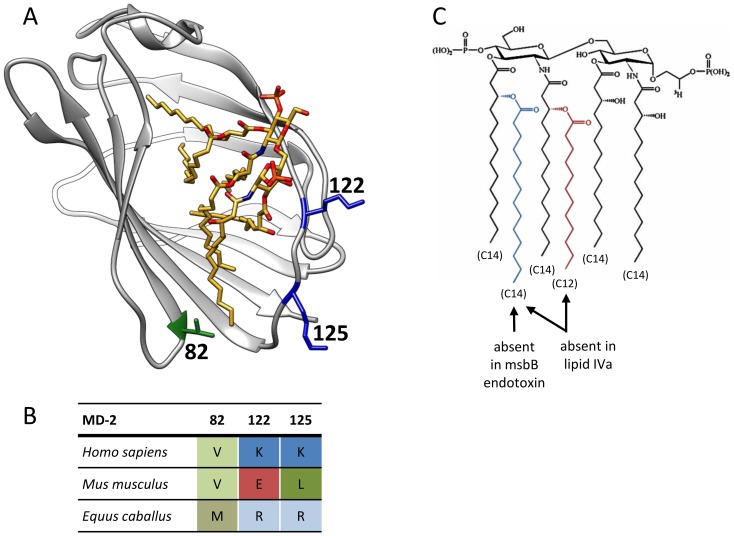
The structure of the human MD-2 with bound endotoxin. (A) Human MD-2 (pdb id 3FXI, [27]) is shown in grey ribbon. The side chains of amino acid residues at positions 82 (valine), 122 (lysine) and 125 (lysine) are shown in stick representation. These amino acid residues are positioned at the entrance to the binding pocket and come in close proximity to the ligand (here the hexaacylated endotoxin, shown in yellow). Figure was prepared with the USCF Chimera package [26]. (B) Amino acid alignment of residues at positions 82, 122 and 125 of human, murine and equine MD-2 (red – acidic amino acid; blue – basic amino acid; green – nonpolar/hydrophobic amino acid). (C) The structure of lipid A. The arrows indicate acyl chains that are absent in the structure of the msbB endotoxin and in lipid IVa.

Since mouse MD-2 enabled activation by lipid IVa in combination with both mouse and equine TLR4, we took a closer look into its tertiary structure in order to identify the amino acid residues responsible for this recognition specificity. We focused on amino acid residues at the rim of the hydrophobic pocket of MD-2 that could come into direct contact with endotoxin and could influence its binding and positioning (Fig. 2, fig. S1). We have previously shown that amino acid residues at positions 122 and 125 markedly affect murine MD-2 solubility and consequently its ability to activate the human TLR4 [31]. These residues are located at a position where they could affect interactions with the phosphate groups of lipid A and influence the positioning of endotoxin into MD-2 and therefore TLR4/MD-2 activation. Residues at positions 122 and 125 have quite different properties between species, including reversed charge in mouse vs. human and equine at position 122 and hydrophobic vs. charged at position 125. We expected that residues at those positions play a decisive role in the selectivity for the tetraacylated lipid IVa. Since the expression levels of TLR4 have been shown to influence the efficiency of endotoxin activation [24], [32], we decided to test all MD-2 mutants on HEK293 cells (T-REx) that stably express TLR4, which closer mimics the physiological conditions than transient transfection of TLR4.

**Figure 3:**
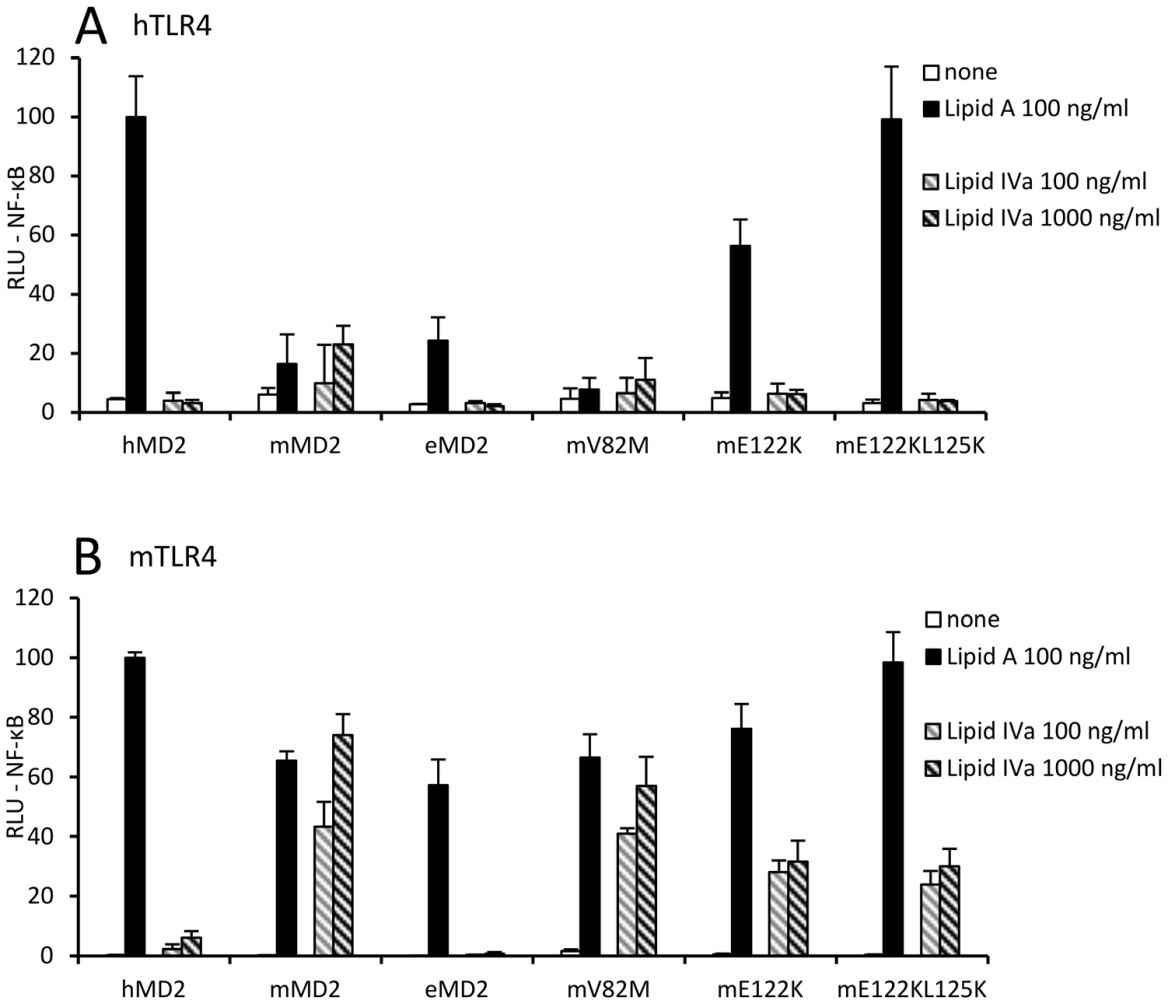
Mutations of murine MD-2 residues E122 and L125 diminish responsiveness to tetraacylated endotoxin while preserving activation by hexaacylated endotoxin. (A) Murine MD-2 mutants mE122K and mE122K L125K gain the ability to activate the human TLR4 with hexaacylated endotoxin. (B) Murine MD-2 mutants mE122K and mE122K L125K in combination with murine TLR4 exhibit reduced activation by tetraacylated lipid IVa.

**Figure 4:**
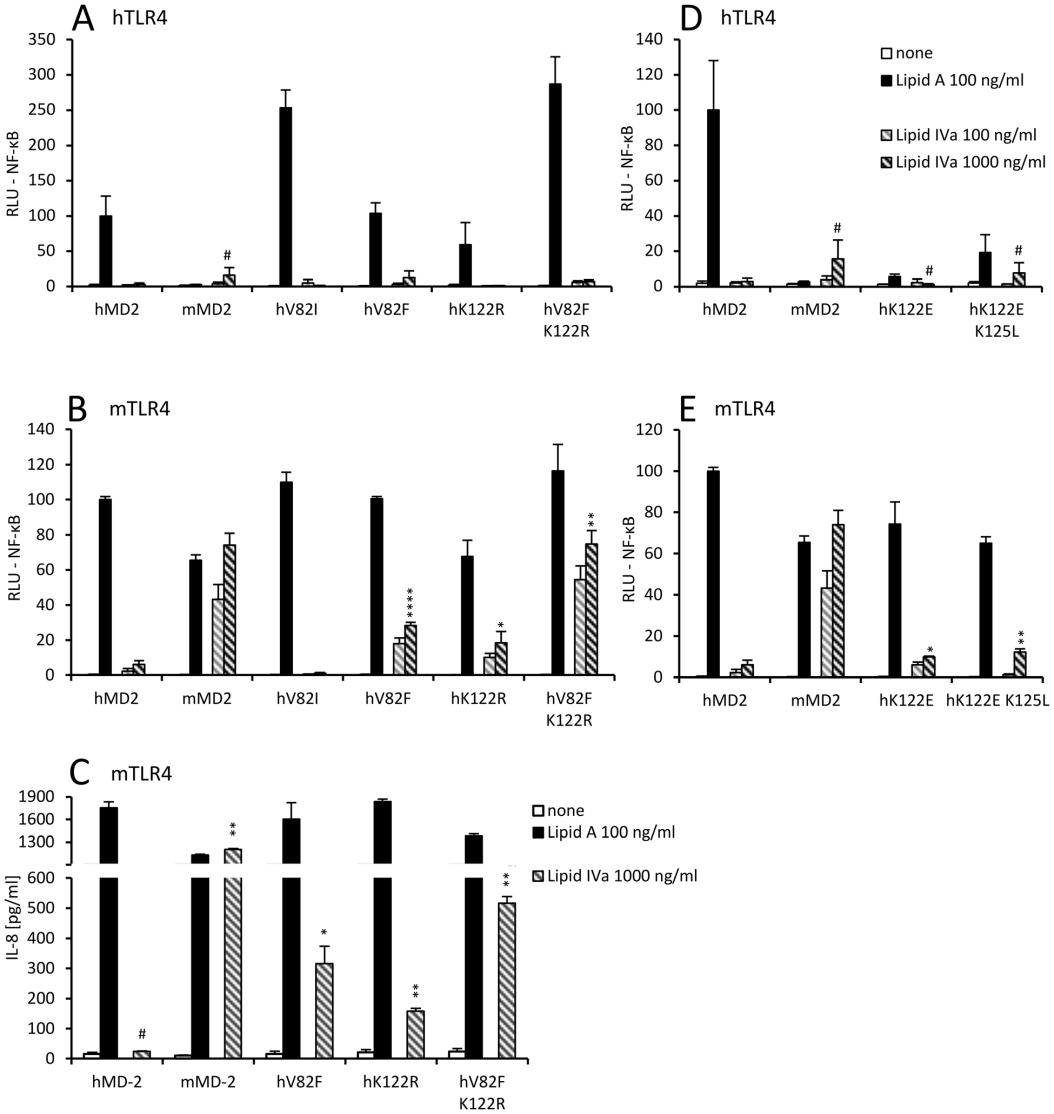
Human MD-2 mutants hV82F and hK122R respond to tetraacylated endotoxin. (A) Human TLR4 does not support activation by tetraacylated endotoxin regardless of MD-2 species or mutations. (B) Specific mutations of human MD-2 enable responsiveness to tetraacylated endotoxin in combination with murine TLR4. (C) Human MD-2 mutants hV82F and hK122R can support IL-8 production by mTLR4-expressing HEK293 cells in response to lipid IVa stimulation. #p>0,1; *p<0,1; **p<0,01 (t-test, compared to mock-stimulated control). (D, E) Mutations of hMD-2 at residues 122 and 125 strongly affect the ability to mediate endotoxin activation. #p>0,1 (not significant); *p<0,1; **p<0,01; ****p<0,0001 (t-test, hMD-2 mutants or mMD-2 stimulated with 1000 ng/ml lipid IVa compared to wt hMD-2 stimulated with 1000 ng/ml lipid IVa).

We found that mutations of mouse MD-2 at positions E122 and L125 markedly reduce its responsiveness to tetraacylated lipid IVa without reducing activation by hexaacylated endotoxin (Fig. 3, fig. S2A), indicating that differences are not due to the solubility of MD-2 mutants or their interactions with the primary binding site of TLR4. Despite the fact that wt mouse MD-2 cannot significantly activate human TLR4, mouse MD-2 mutants mE122K and mE122K L125K, whose amino acid residues are replaced with the corresponding residues of human MD-2, gained the ability to potently activate human TLR4 in response to hexaacylated endotoxin stimulation (Fig. 3A and [31]), but remained unresponsive to lipid IVa in combination with human TLR4 (Fig. 3A). In the proximity of the amino acid residue 122 on MD-2 there is a hydrophobic loop between amino acid residues 82 and 87 that is important for activation by hexaacylated endotoxin [33]. We reasoned that this region of MD-2 could be an additional candidate for the selectivity. Residues in this hydrophobic loop are highly conserved between species. One of the rare exceptions is the equine MD-2, which has similar residues at positions 122 and 125 as human MD-2, but has a methionine amino acid residue at position 82 instead of a much more frequent valine. Since the equine TLR4/MD-2 complex also supports activation by lipid IVa, we anticipated that V82M substitution would influence the activation by lipid IVa. However, the mouse MD-2 mutant mV82M in combination with mouse TLR4 showed no significant change in activation by either hexa- or tetraacylated forms of endotoxin (Fig. 3B). Also with human TLR4 there were no significant differences in the responsiveness to different endotoxins when comparing the mV82M mutant with wild type mMD-2. We then investigated the role of the same residues in human MD-2 (Fig. 4, fig. S2B). None of the human MD-2 mutants tested managed to trigger activation by lipid IVa on human TLR4 expressing cells (Fig. 4A).

Nevertheless, based on the tertiary structure we deduced the contribution of the hydrophobic interactions involving the amino acid residue at position 82 in MD-2 and the acyl chain of the lipid A. We hypothesized that introduction of a bulky hydrophobic residue at this position should improve activation by hypoacylated lipid A. Indeed, we found that substitution of the valine at position 82 of human MD-2 with a bulkier hydrophobic phenylalanine (but not with a hydrophobic residue with only slightly larger side chain, *i.e*. isoleucine) markedly improved activation by lipid IVa in combination with mTLR4. This activation was further enhanced in the case of a double mutant (hMD-2_V82F K122R), where we combined the effect of this position with the corresponding 122 residue of equine MD-2, reaching the level of activation by lipid IVa comparable to the wild type murine MD-2 (Fig. 4B and C).

The amino acid substitution at position 122 of MD-2 of lysine with arginine, which retains the cationic charge, is one of the rare differences between the amino acid residues surrounding the hydrophobic pocket of human and equine MD-2 (Fig. 2B). Nonetheless equine MD-2 (in combination with eTLR4) enabled activation by lipid IVa in contrast to human MD-2 (Fig. 1). Surprisingly we found that this subtle mutation (hMD-2_K122R) markedly augmented activation of mouse TLR4 in response to lipid IVa stimulation (Fig. 4B).

Though the charge reversal in human MD-2 mutants hK122E and hK122E K125L impaired activation by lipid A of cells expressing human TLR4, probably due to the reduced electrostatic interactions with the phosphate group of lipid A (Fig. 4D and [31]), these mutants gained significant responsiveness to lipid IVa stimulation in combination with mouse TLR4 (Fig. 4E).

**Figure 5:**
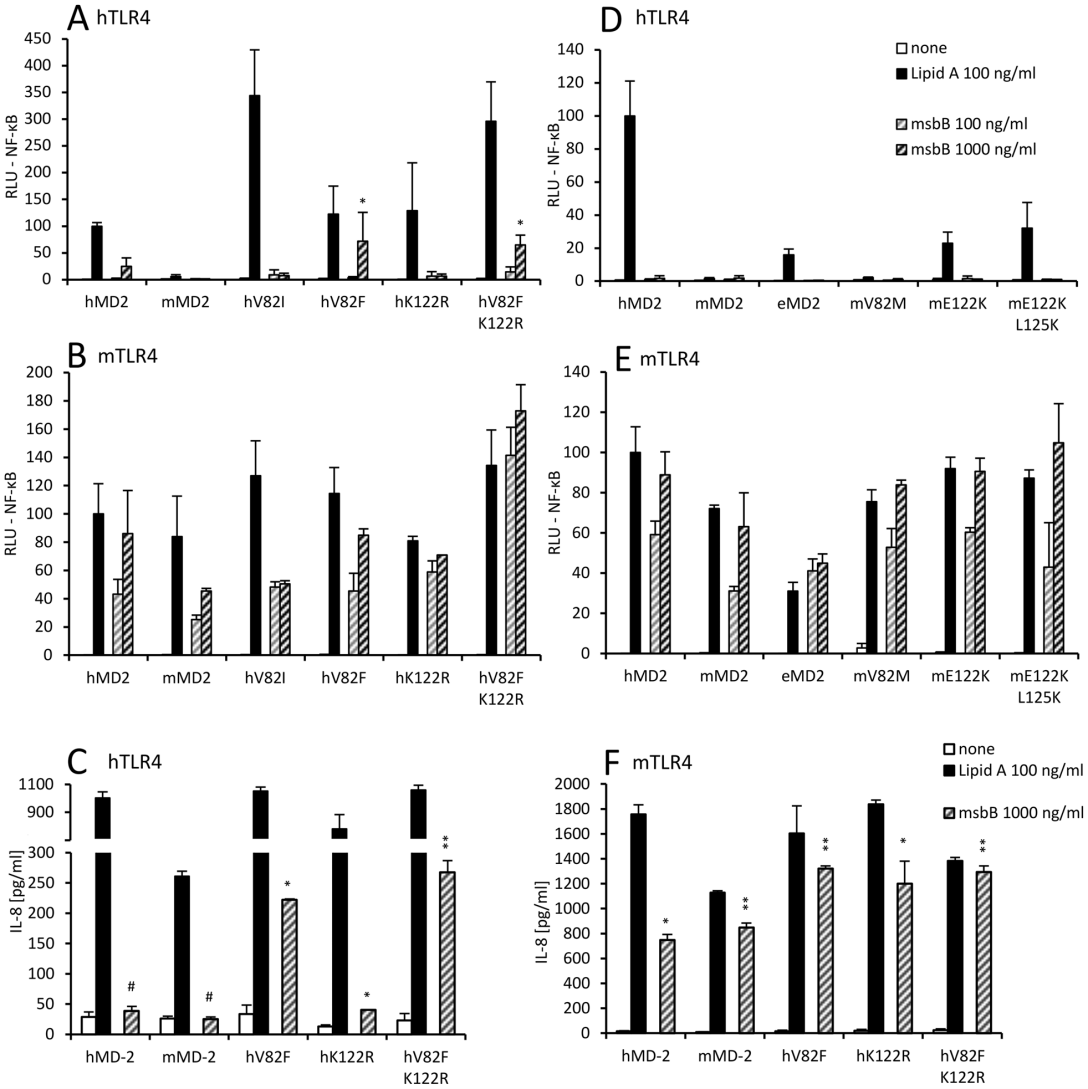
Human MD-2 mutants exhibit activation by pentaacylated endotoxin and bypass the requirement for murine TLR4. (A) Human MD-2 mutants hV82F and hV82F K122R enable activation of human TLR4 by pentaacylated endotoxin. *p<0,5; **p<0,05 (t-test, compared to wt hMD-2 stimulated with 1000 ng/ml msbB). (B) Human MD-2 (mutants and wt) support activation of mouse TLR4-expressing cells by pentaacylated endotoxin. (C) Human MD-2 mutants hV82F and hK122R can support IL-8 production by hTLR4-expressing HEK293 cells in response to msbB stimulation. (D) Mouse MD-2 mutants that respond to hexaacylated lipid A stimulation do not gain responsiveness to pentaacylated endotoxin in combination with human TLR4. (E) No msbB responsiveness is lost with mouse MD-2 mutants when in combination with mouse TLR4. (F) Murine TLR4 supports potent IL-8 production regardless of the MD-2 species. (C, F) #p>0,1; *p<0,1; **p<0,01 (t-test, compared to mock-stimulated control).

TLR4 activation by pentaacylated lipid A, however, does not follow the pattern of activation by tetraacylated lipid A. Therefore we next investigated whether the amino acid residues at positions 82 and 122 of MD-2 affect its ability to trigger activation by pentaacylated endotoxin in the same manner as with tetraacylated lipid IVa (Fig. 5, fig. S2C and D). We found that introduction of a large hydrophobic residue at position 82 (*i.e*. hMD-2 mutant V82F) is already sufficient to render human MD-2 responsive to pentaacylated msbB endotoxin (Fig. 5A and C). On the other hand, amino acid substitution by a residue with slightly larger hydrophobic side chain (*i.e.* hMD-2 mutant V82I) had no effect. Since the equine MD-2 robustly responds to msbB in combination with mouse or equine TLR4 (Fig. 1), we mutated the lysine at position 122 into arginine that is found in equine MD-2 at this position to investigate its potential role in this context. However, this substitution had no effect on the activation of human TLR4 by pentaacylated endotoxin either (Fig. 5A).

We next analyzed which mouse MD-2 mutants mediated hexaacylated lipid A recognition in combination with human TLR4. None of these mMD-2 mutants (*i.e.* mE122K, mE122K L125K) mediated pentaacylated msbB-driven cell activation via the hTLR4 (Fig. 5D). These mMD-2 mutants, which have amino acid substitutions at positions that are important for signaling, did not reduce the ability of mouse TLR4 to respond to pentaacylated endotoxin (Fig. 5E). Indeed, it seems that mouse TLR4 and not mouse MD-2 has a decisive role in this selectivity, since mouse TLR4 enables activation by pentaacylated msbB regardless of the MD-2 variety (Fig. 5B and F).

## Discussion

We have detailed knowledge of the structure of the activated human and mouse TLR4/MD-2/endotoxin receptor complexes. Since the positioning of endotoxin into MD-2 and its interaction with TLR4 ectodomain is governed by a complex combination of electrostatic as well as hydrophobic interactions, the precise role of the residues mediating the species-specific TLR4/MD-2 activation by hypoacylated endotoxin varieties is still not clear. In the present study we addressed this question by comparing the capacity of MD-2 and TLR4 from three species to activate cells in response to hypoacylated endotoxin stimulation.

We found that despite high overall sequential and structural similarity between human, mouse and equine endotoxin receptors the capability of triggering potent cellular activation varies when combining MD-2 and TLR4 from different species, indicating complex balance of interactions (Fig. 1). Combining MD-2 and TLR4 molecules from different species gives us an opportunity to gain further insight into these interactions. Our results show that although human MD-2 renders cells responsive to hexaacylated lipid A regardless of TLR4 species, it is completely unable to mediate activation by lipid IVa (Fig. 1). On the other hand, the tetraacylated endotoxin potently activates both wt equine and mouse TLR4/MD-2. Intriguingly, while mouse MD-2 can activate cells in response to lipid IVa stimulation in combination with either mouse or equine TLR4, equine MD-2 can do the same only in combination with equine TLR4 and not with mouse TLR4. Our results therefore indicate that among the investigated MD-2 species, the mouse MD-2 is best adapted for activation by tetraacylated lipid IVa, since it mediates potent cell activation in combination with wt mouse as well as equine TLR4.

In case of lipid IVa, MD-2 seems to be the key player determining the species-specificity of tetraacylated endotoxin recognition (Fig. 1, [23], [34]). On the other hand, TLR4 appears to play a more important role in the recognition of pentaacylated msbB endotoxin, as mouse TLR4 enables cellular activation in response to msbB regardless of the origin of MD-2 (Fig. 1). Even though msbB endotoxin antagonizes the ability of hexaacylated endotoxin to stimulate human endothelial cells [10], [35] as well as HEK293 cells transfected with human TLR4/MD-2 [32], we show here that it can potently activate mouse and equine TLR4/MD-2 receptor complex. This suggests that pentaacylated endotoxin can bind to MD-2 yet this complex is able to trigger activation of only mTLR4 but not of hTLR4 (resulting in an antagonistic effect). The number and position of phosphate groups of lipid A was also reported to be important for the agonistic properties of pentaacylated endotoxins with, interestingly, 1-phosphorylated pentaacylated msbB endotoxin having higher human TLR4/MD-2 stimulatory properties than bi-phosphorylated msbB [36]. Our findings, *i.e.* that both partners in the TLR4/MD-2 complex contribute to the species-specific recognition of endotoxin, are supported by the observation that the TLR4/MD-2 complex has higher affinity for endotoxin than MD-2 by itself [37].

Recently crystal structures of two complexes of mouse TLR4 and MD-2 were determined, one with bound Re-LPS and one with lipid IVa [38]. They provided for the first time a detailed insight into the mouse TLR4/MD-2 receptor in complex with an agonist. Interestingly, they revealed that the position of lipid IVa in the hydrophobic pocket of mouse MD-2 is inverted by approximately 180 degrees compared to how it binds into the human MD-2 [28], [38]. The reason behind this change in lipid IVa positioning lies in the amino acid residues that differ between the mouse and the human TLR4/MD-2. Several mutational studies tried to pinpoint these amino acid differences, but could not explain why lipid IVa acts as an agonist in some and as an antagonist in other species. Notably, K367 and R434 of mouse TLR4, which are not conserved in human TLR4, were suggested to influence the binding of lipid IVa through interactions with 1-PO_4_ group of the glucosamine backbone [25], [38]. Interestingly, equine TLR4, which is capable of mediating lipid IVa activation with the help of either mouse or equine MD-2 (Fig. 1C), has glutamic acid and glutamine at positions 367 and 434, respectively, which are the same amino acid residues as in human TLR4. It is therefore clear that a cooperative interaction of several amino acid residues determines the outcome of lipid IVa activation. We and others have shown a significant influence of the amino acid residues E122 or K122 in mouse or human MD-2, respectively [25], [31]. We observed a significant reduction of the lipid IVa activation in the case of mMD-2_E122K mutant in combination with mTLR4. Even though this mutant gained the ability to activate human TLR4 in response to lipid A (Fig. 3, [31]), it still remained unresponsive to lipid IVa in combination with human TLR4, emphasizing an important discriminatory role not only of MD-2, but also of TLR4. Moreover, we included the double mutant mMD-2_E122K L125K in our experiments to determine, if the second mutation (which further improves solubility and the ability of the mutant to support activation by canonical hexaacylated lipid A in combination with hTLR4 [31]), permits activation with hypoacylated endotoxins in combination with hTLR4. This turned out not to be the case (Fig. 3A), which suggests that the role of the positively charged lysine 125 in hMD-2 is solely in increased solubility of the protein and not in the positioning of the endotoxin into the hydrophobic binding pocket.

**Figure 6:**
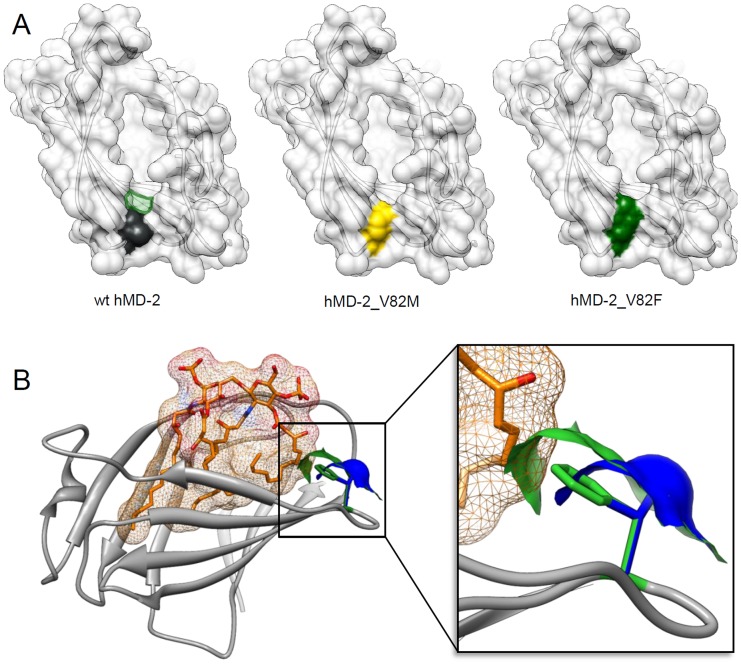
Large amino acid residue side chains at position 82 in hMD-2 augment hydrophobic interactions and promote TLR4 activation by hypoacylated endotoxins. (A) The surface representation of human MD-2 (2E56, [28]) and mutants at position 82. (left) wt hMD-2, (middle) hMD-2_V82M, (right) hMD-2_V82F. The surface at the amino acid residue at position 82 of wt MD-2 and both mutants is colored in grey, yellow or green, respectively. Green translucent area on the wt MD-2 (left) represents the difference in surface area occupied in the hMD-2_V82F mutant. (B) The structure of human MD-2 (2E59 [28]) with bound lipid IVa. At position 82 side chains and the corresponding molecular surfaces are shown for the wt hMD-2 valine (blue) and phenylalanine mutant (green). Introduction of a large phenylalanine 82 side chain causes clash with a buried acyl chain of lipid IVa (right, close-up view). Figures were prepared with the USCF Chimera package [26].

Intriguingly although mutation of the basic lysine 122 in human MD-2 into the acidic glutamic acid increases the ability of human MD-2 to confer lipid IVa activation at least in combination with mouse TLR4 (Fig. 4), this amino acid difference cannot explain why equine TLR4/MD-2 supports activation by lipid IVa. Equine MD-2 has a basic arginine residue at position 122 and also an overall surface charge distribution more similar to human MD-2. It is reasonable to assume that changes in the electrostatic potential at the hydrophobic pocket entrance could influence the interactions with charged phosphate groups of the lipid IVa and thus indirectly influence the agonistic vs. antagonistic orientation of the lipid IVa. Our study demonstrates that the contribution of not only the electrostatic charge but also of the hydrophobicity and the size of specific amino acid side chains influences the agonistic effect of hypoacylated endotoxins. We show that an increased size of the amino acid side chain at position 82 of human MD-2 (*i.e.* hMD-2_V82F) markedly improves its ability to activate mouse TLR4 expressing cells in response to lipid IVa stimulation (Fig. 4B and C). The hMD-2_V82F mutant responded to pentaacylated msbB even in combination with human TLR4 (Fig. 5A). A large amino acid side chain at this specific position, which is right at the very base of the entrance into the hydrophobic pocket, narrows this entrance and can obstruct the accommodation of all five endotoxin acyl chains (Fig. 6); a phenylalanine residue at position 82 of MD-2 (instead of a valine residue) would decrease the accessible entrance to the hydrophobic pocket by approximately 2,8 Å or 12%. This could force one of the acyl chains to remain outside the MD-2 binding pocket and mediate the interaction with TLR4 that drives receptor activation. Indeed, canine MD-2 has phenylalanine at position 82 and equine MD-2 has a similarly large methionine that could contribute to their ability to respond to lipid IVa stimulation regardless of their otherwise closer similarity to the unresponsive human MD-2. On the other hand, amino acid with a small side chain, such as alanine, has a profoundly negative effect even on signalization with hexaacylated endotoxin [39]. A comparison of the size of the MD-2 binding pockets revealed that the smallest pocket (1690 Å^3^) is formed by the human MD-2 and the largest (2100 Å^3^) by the equine MD-2, while the size of the murine MD-2 binding pocket (1920 Å^3^) is in between. Therefore in the case of the eMD-2, a narrower entrance to the binding pocket could increase the tendency of one endotoxin acyl chain to remain outside of the pocket, leading to receptor activation.

In conclusion, our study provides a systematic comparison of the human, mouse and equine TLR4/MD-2 receptor complexes and offers further insight into the agonistic/antagonistic response to two different hypoacylated endotoxin varieties. It surprisingly reveals that although mouse and equine TLR4/MD-2 receptor complexes both support activation by tetraacylated lipid IVa, mouse MD-2 can respond to lipid IVa stimulation even with equine TLR4, but not the reverse combination. Moreover, mouse TLR4 supports activation by pentaacylated msbB regardless of the MD-2 species, underlining the importance of both MD-2 as well as TLR4 in species-specific recognition of different endotoxins. We have demonstrated the gain of responsiveness to hypoacylated lipid A by hMD-2 mutants, showing that it is relatively easy to modify the endotoxin chemotype specificity. Since each organism encounters a specific array of pathogens, each species has probably acquired the degree of responsiveness that strikes a balance between the detection of pathogens and excessive activation of the innate immune response.

## Supporting Information

Figure S1 **Amino acid alignment of the MD-2 co-receptor from three different species, i.e. human, mouse and horse.** The dots in the alignment designate identical amino acid at a specified position. The color of the letters depict chemical properties of individual amino acid residues (red – acidic; blue – basic; green – polar, uncharged; black – nonpolar (hydrophobic)).
(TIF)

Figure S2 **Activation of the equine TLR4 receptor by hypoacylated endotoxins.** (A) Mutations of murine MD-2 residues E122 and L125 diminish responsiveness to tetraacylated endotoxin. (B) Human MD-2 mutants hV82F and hK122R respond to tetraacylated endotoxin. (C) Only the wt eMD-2 exhibits potent activation by pentaacylated endotoxin in combination with equine TLR4. (D) Human MD-2 mutants hV82F and hK122R respond to pentaacylated endotoxin in combination with equine TLR4.
(TIF)
