# Supplementary figures and images for: Species-Specific Activation of TLR4 by Hypoacylated Endotoxins Governed by Residues 82 and 122 of MD-2
Source: PLoS One. 2014 Sep 9;9(9):e107520. doi: 10.1371/journal.pone.0107520 (PMC4159346; doi:10.1371/journal.pone.0107520)

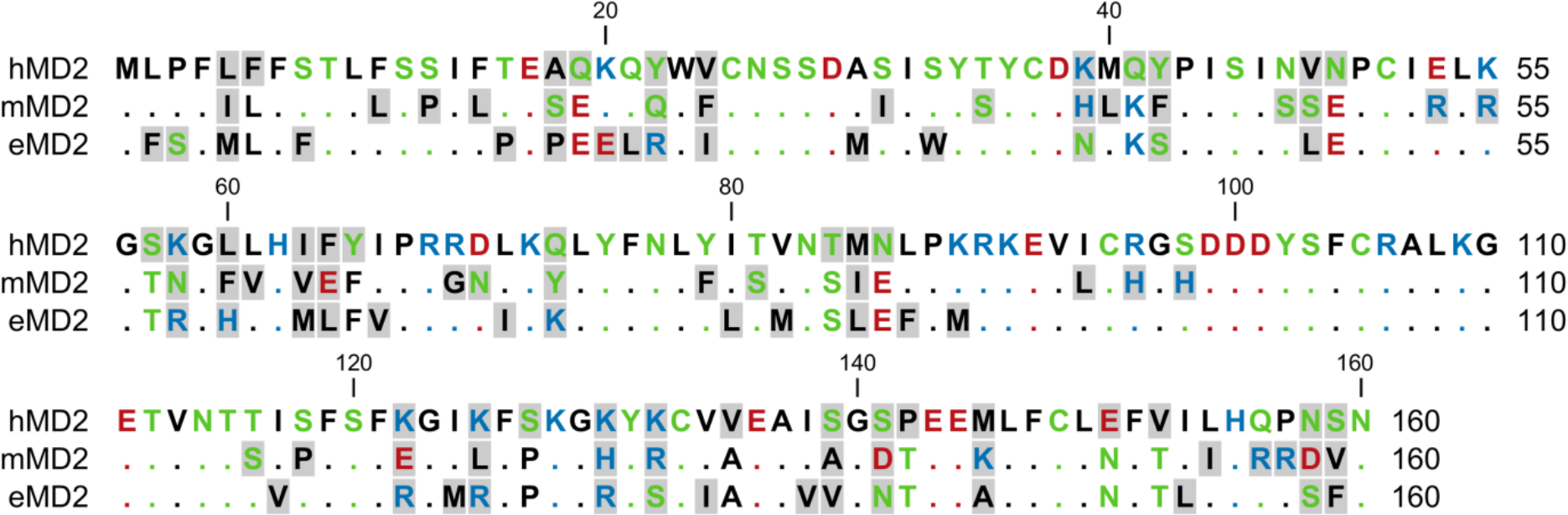

Supplement: Figure S1 — Amino acid alignment of the MD-2 co-receptor from three different species, i.e. human, mouse and horse. The dots in the alignment designate identical amino acid at a specified position. The color of the letters depict chemical properties of individual amino acid residues (red – acidic; blue – basic; green – polar, uncharged; black – nonpolar (hydrophobic)). (TIF) [file pone.0107520.s001.tif]
